# Hypereosinophilic vasculitis with Raynaud phenomenon presentation: a case report

**DOI:** 10.1186/s43044-023-00418-6

**Published:** 2023-10-18

**Authors:** Duy Le Cao Phuong, Hoa Bui The, Quan Vo Duy

**Affiliations:** 1 Department of Cardiovascular Intervention, Nguyen Tri Phuong Hospital, Ho Chi Minh City, Vietnam; 2 Faculty of Medicine, Nguyen Tat Thanh University, Ho Chi Minh City, Vietnam

**Keywords:** Eosinophilic disorders, Polyangiitis, Eosinophilic vasculitis, Hypereosinophilic syndrome, Corticosteroid

## Abstract

**Background:**

Previous case series have reported idiopathic eosinophilic vasculitis as a potential manifestation of hypereosinophilic syndrome (HES). This condition is characterized by digital necrotizing, systemic vasculitis that affects varying-sized blood vessels. This report presents our experience in treating a patient with eosinophilic vasculitis.

**Case presentation:**

We describe the case of a 23-year-old man who presented with idiopathic HES, which manifested as digital ulcers and peripheral ischemia in both the upper and lower limbs, without the involvement of other organ systems. After ruling out primary and secondary causes of eosinophilia, a diagnosis of HES was established. Our patient has shown a positive response to corticosteroid therapy.

**Conclusions:**

Our case contributes to the existing evidence about diagnosing idiopathic eosinophilic vasculitis in patients with HES. We observed a favorable response to corticosteroid treatment in our patient.

## Background

Hypereosinophilia is defined as an absolute eosinophil count exceeding 1500/L. It can occur as a response to infections, parasitic infestations, or malignancies [1]. Hypereosinophilia can cause damage to various organs, including the skin, heart, lungs, and central and peripheral nervous systems.

Hypereosinophilic vasculitis (HES) is an entity associated with hypereosinophilic syndromes characterized by vascular injury. This condition carries an increased risk of thrombosis in both veins and arteries, which can be life-threatening. According to a study in the UK, the incidence of HES ranged from under 0.04–0.17 per 100,000 person-years, and its prevalence ranged from 0.15 to 0.89 cases per 100,000 persons [2]. Hypereosinophilic vasculitis affects blood vessels of varying sizes, predominantly small vessels but also medium and large vessels.

The proposed pathophysiology revolves around the direct toxic effects of activated eosinophils and their extracellular granules, which contribute to necrosis of the vascular walls in smaller- to medium-sized vessels. In some cases, the condition can also lead to arterial dissection and aneurysms in medium- to large-sized vessels [3]. However, the ischemic symptoms observed in this case resembled those of thromboangiitis obliterans (TAO), a rare condition with distinct characteristics.

Takegawa et al. reported a case of an individual with idiopathic hypereosinophilic syndrome presented with digital necrosis. They proposed that tumor necrosis factor (TNF) may play a role in the underlying mechanism [4]. In addition, CD40, a receptor for a 35-kDa glycoprotein known as CD40L and a member of the TNF-α superfamily, is expressed by T cells, mast cells, and basophils. CD40L has been found to be involved in the regulation of B-cell growth, immunoglobulin class switching, and the activation of monocytes and T cells. In vitro, experiments have shown that eosinophils, when combined with IL-4, can trigger CD40L-dependent B-cell proliferation [5].

The present case involves a unique presentation of vasculitis affecting medium- to small-sized blood vessels accompanied by hypereosinophilia. The antineutrophil cytoplasmic antibodies (ANCA) were negative, and there is no evidence of respiratory tract injury or the presence of glomerulonephritis.

## Case presentation

A 23-year-old man presented to our department with cyanosis in both hands and a painful ulcer on the second and third fingers of his right hand, which had appeared 5 days prior. He also reported experiencing paresthesia in his toes and intermittent claudication in his lower limbs for the past 2 months.

There was no history of fever, respiratory symptoms, or oral or genital ulcers. Additionally, no history of autoimmune disease was found in his family history, and he had never smoked and denied any drug involvement.

**Fig. 1:**
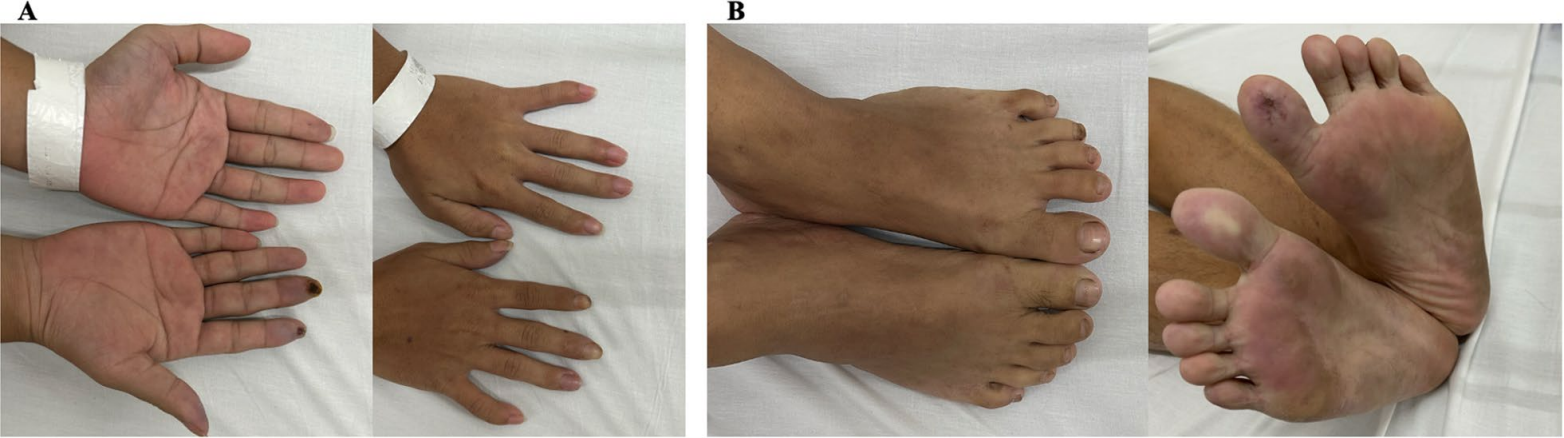
The patient’s ischemic manifestations with digital infarctions. **A** Ischemic manifestations of both hands, gangrenous changes localized to the second and third fingers of the right hand. **B** Ischemic signs in the lower extremities with concomitant gangrenous changes specifically affecting the first toe of the left leg

During the physical examination, his body temperature was within the normal range, and his blood pressure was normal in both his upper and lower limbs, showing no noticeable difference. His distal fingers appeared cold and slightly cyanotic, with painful ulcers and infarction on the right hand's second and third fingers. The reduced capillary filling was observed in all fingers of his upper limbs. Peripheral pulse examination revealed intact ulnar and radial pulses, but the dorsalis pedis pulses were absent (Fig. 1).

There were no visible erythematous, angioedematous, urticarial lesions, pruritic papules, or nodules at the time of presentation. Other systemic examinations, including musculoskeletal, respiratory, ocular, cardiac, and abdominal evaluations, revealed no significant findings.

The complete blood count results revealed a total white blood cell count of 17.5 K/μL (normal range: 4–10), with an absolute eosinophil count of 4.9 K/μL (normal range: 0.01–0.8). Hemoglobin level was measured at 13.3 g/dL (normal range: 12–15), and the platelet count was 164 K/μL (normal range: 150–400). The C-reactive protein level and erythrocyte sedimentation rate were within normal ranges. Additionally, the levels of creatinine, liver function markers, and results of urinalysis and hsTroponin I did not show any abnormalities. Baseline prothrombin time and activated partial thromboplastin time were normal, although the D-dimer level was elevated. Comprehensive infection screening tests, including HIV, hepatitis B and C viruses, Strongyloides, and Toxocara, all returned negative results. Stool tests for ova and parasites were also negative.

Further immune serology screening was performed to explore potential underlying factors contributing to hypereosinophilia. The results indicated negative findings for p-ANCA, c-ANCA, anti-B2 glycoprotein, antinuclear antibody, anti-double-stranded DNA, lupus anticoagulant, rheumatoid factor, anti-phospholipid panel, and anticardiolipin. Levels of complement (C3, C4) and immunoglobulins were within normal ranges. Genetic testing was conducted to investigate possible heterogeneous causes, but no FIP1L1/PDGFRA gene fusion was detected. Immunophenotyping of T-cell and T-cell receptor rearrangement studies were not conducted due to limitations in genetic and laboratory testing in Vietnam.

**Fig. 2:**
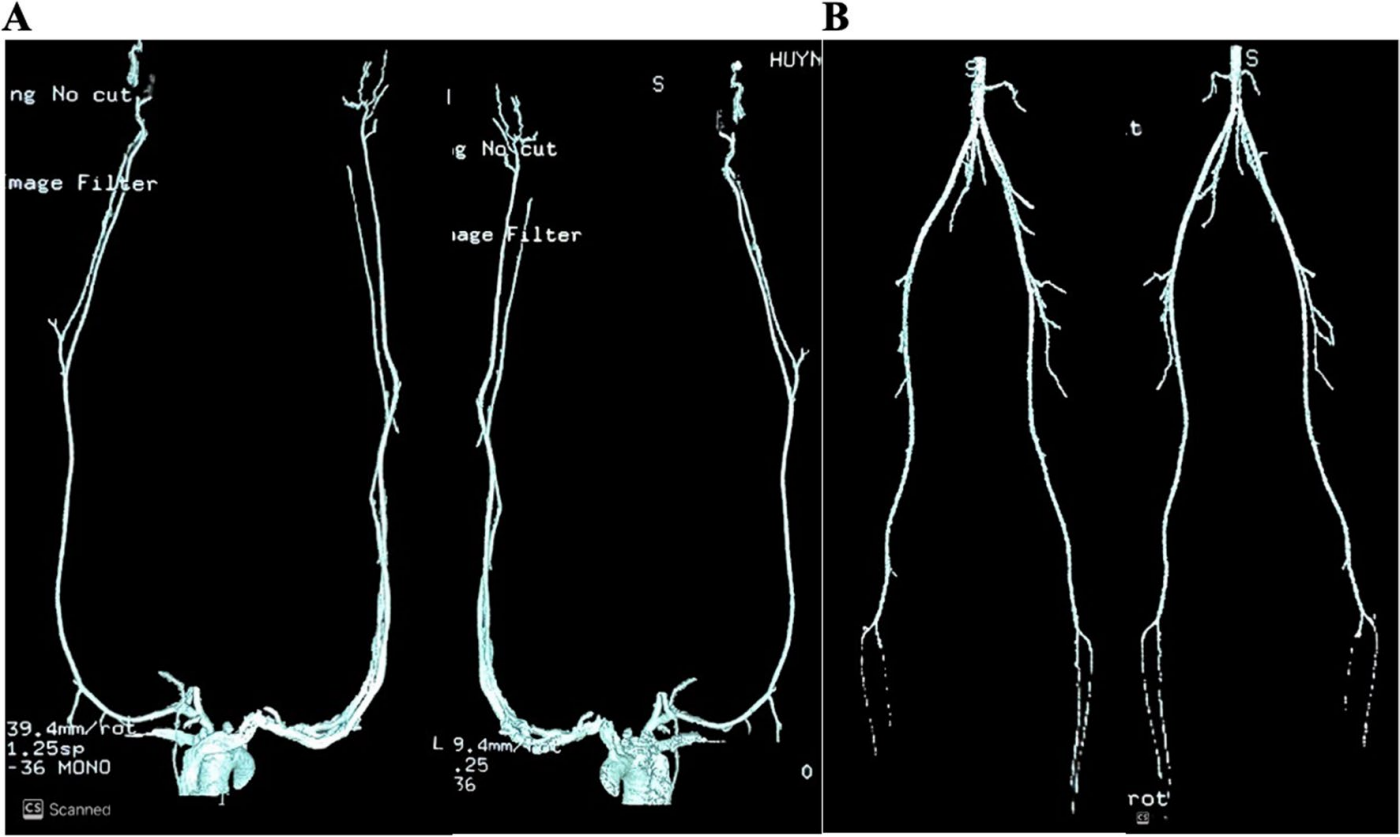
Extremity angiography on computed tomography. **A** Normal angiography of the upper limbs. **B** Occlusion of the anterior and posterior tibialis arteries without evidence of atherosclerosis

A transthoracic echocardiogram revealed no signs of vegetations, valve involvement, or abnormalities in heart wall motion. Abdominal sonography yielded normal results. Electromyography indicated severe bilateral polyneuropathy in the lower limbs. However, lumbar and sacral spine MRI showed no evidence of spinal cord disease. Doppler ultrasonography identified arterial thrombosis affecting anterior and posterior tibialis arteries of both lower extremities, and these findings were confirmed by CT angiography (Fig. 2).

Skin biopsy was not conducted mainly because of apprehensions regarding ischemic-induced wound healing impairment in the gangrene lesion. The patient received aspirin and heparin treatment upon admission, so bone marrow biopsy was not indicated, primarily due to concern about the potential risk of significant hemorrhagic complications.

**Fig. 3:**
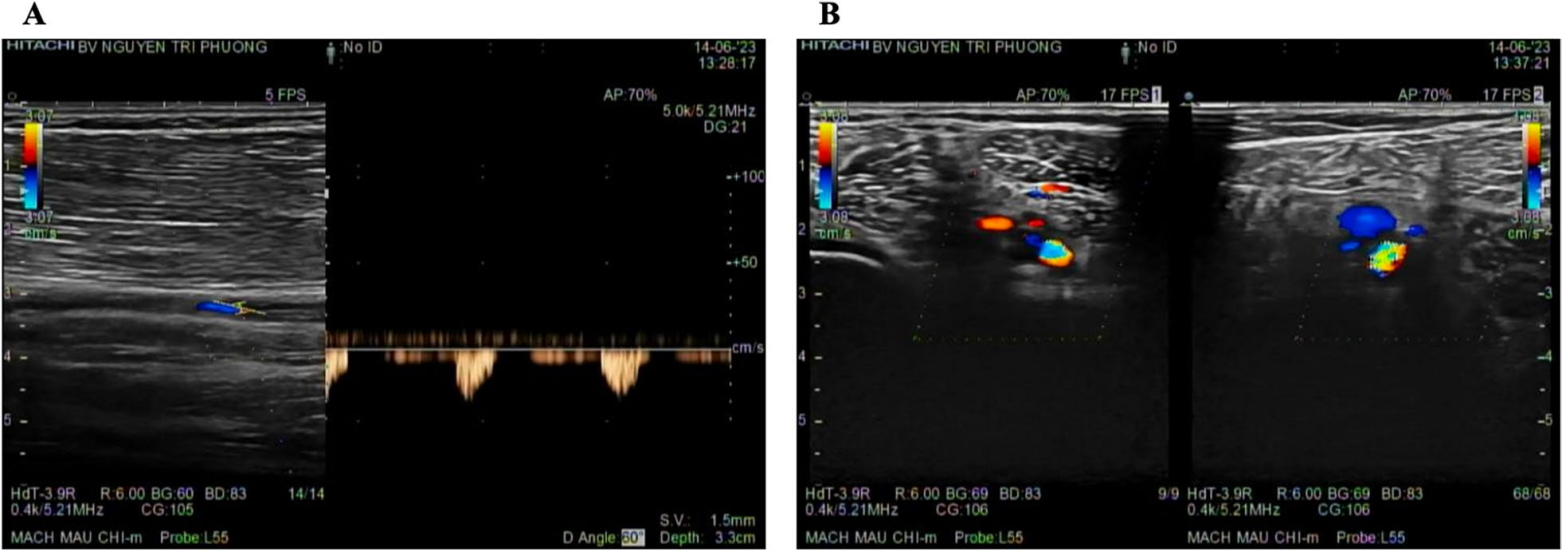
Doppler ultrasound of lower extremity’s vascular after 2 weeks of treatment. **A** Images of single-phase waveforms in the anterior and posterior tibialis arteries. **B** Thrombosis-induced partial occlusion of the tibialis arteries

The most likely diagnosis of small and medium vessel vasculitis induced by hypereosinophilic syndrome (HES) was established. Although the subtype of HES could not be confirmed in this case, we decided to commence corticosteroid treatment, which is considered first-line therapy in most forms of HES. To initiate treatment. We used immunosuppressant therapy with methylprednisolone at a dosage of 1 mg/kg/day for 2 weeks. Additionally, anticoagulation treatment was continued using low molecular weight heparin. The patient was also prescribed aspirin at a daily dosage of 81 mg and atorvastatin at a daily dosage of 20 mg. This treatment regimen resulted in significant improvements both clinically and in laboratory parameters. The patient reported a noticeable reduction in digital pallor and improvement in intermittent claudication symptoms. Upon physical examination, the patient's distal pulses in the lower limbs were now detectable. The eosinophil level decreased from over 4.9 to 0.9 K/μL (Table 1). However, the lower-limb arterial Doppler scan performed at discharge indicated ischemia evidence with the image of monophasic flow and thrombosis in the anterior and posterior tibialis arteries of both legs (Fig. 3).

**Table 1:** Patient eosinophil count during follow-up

	At presentation	At 2 weeks (hospital discharge)	1 month after discharge	2 months after discharge
WBC (K/μL)	17.5	13.8	15.3	12.6
Eosinophils (%)	28	25	3.5	2.1
Eosinophils (K/μL)	4.9	0.9	0.5	0.3
Treatment	Methylprednisone 1 mg/kg/day	Methylprednisone 1 mg/kg/day	Prednisone 20 mg/day	Prednisone 10 mg/day

Following 2 weeks of treatment during hospitalization, the patient was discharged and prescribed methylprednisolone at a daily dosage of 1 mg/kg/day. Additionally, the patient was scheduled for regular follow-up appointments at our outpatient clinic.

## Discussion

**Fig. 4:**
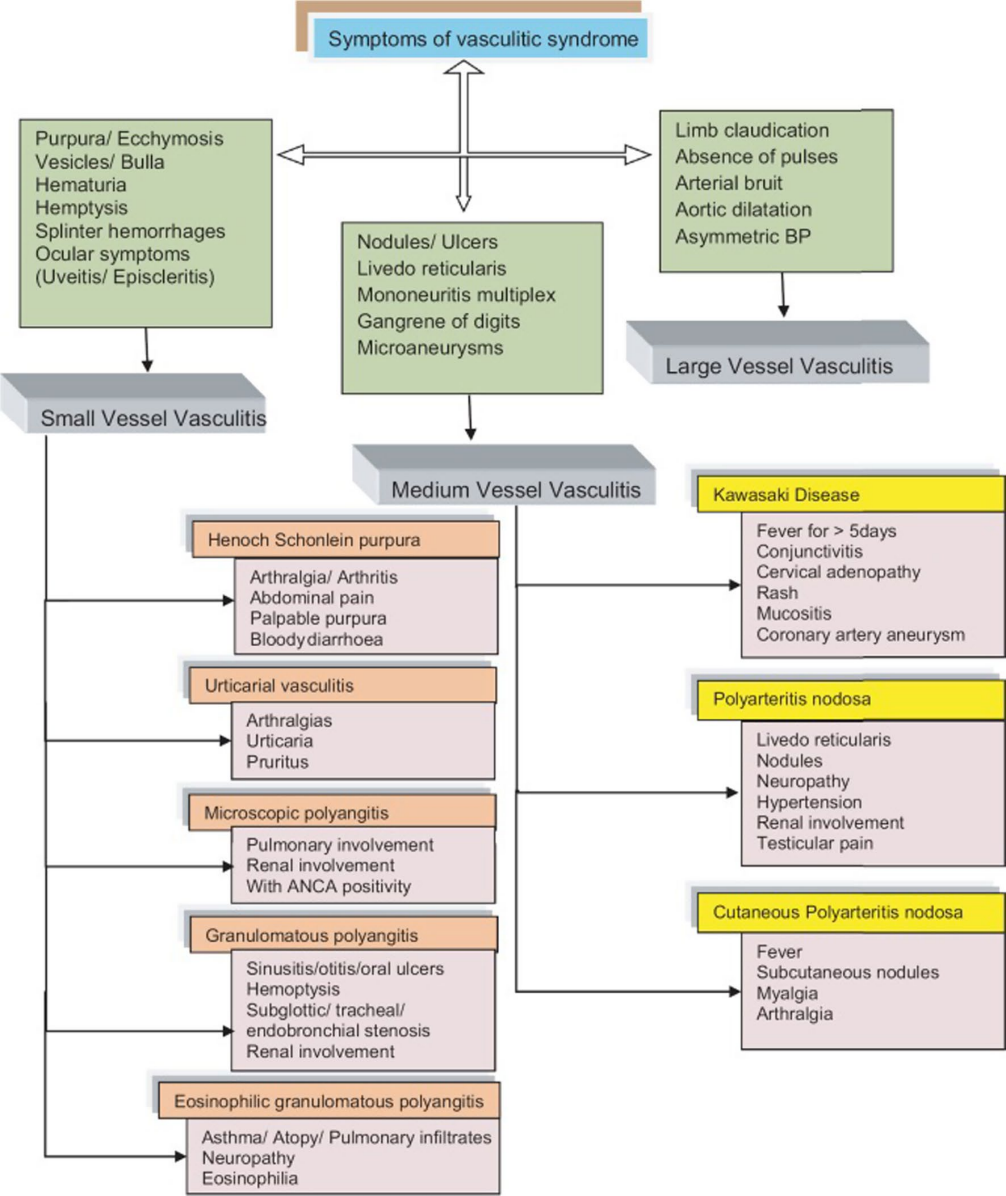
Algorithm for the syndromic approach of symptoms in vasculitis adapted Sangolli, P.M., and Lakshmi, D.V., Vasculitis: a checklist to approach and treatment update for dermatologists. Indian Dermatol Online J, 2019. 10(6): p. 617–626

**Fig. 5:**
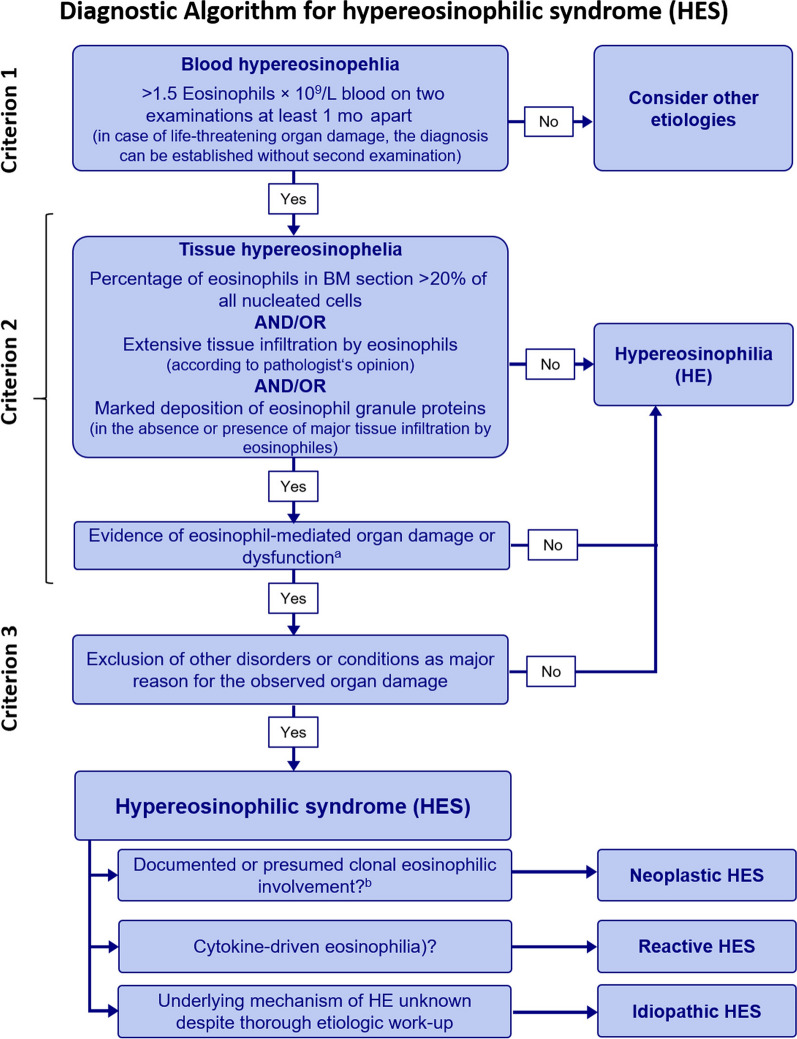
Diagnostic algorithm for hypereosinophilic syndrome adapted Schuster, B., Zink, A., and Eyerich, K., Medical algorithm: diagnosis and treatment of hypereosinophilic syndrome. Allergy, 2020. 75(11): p. 3003–3006

Our patient presented with a distinct manifestation of the Raynaud phenomenon, characterized by digital infarction in two fingers and elevated eosinophil levels, without respiratory or renal system involvement. The presence of the Raynaud phenomenon and obstruction in the bilateral tibialis artery observed in the CT angiography suggests a possible involvement of medium to small vessels, raising the consideration of Thromboangiitis obliterans (Buerger's disease). However, Buerger's disease is not typically associated with eosinophilia. Another potential consideration is eosinophilic granulomatosis with polyangiitis (Churg–Strauss syndrome), although it appears less likely due to the absence of pulmonary involvement and negative immunologic screening results. Secondary vasculitis is commonly associated with infections, drug usage, or rheumatic diseases. However, in our patient's case, the manifestation involved both medium and small vessels, which is distinct from the typical primary small vessel involvement seen in secondary vasculitis. Additionally, the laboratory did not support any possible causes of this entity. After ruling out viral causes of vasculitis, the most probable diagnosis aligns with a distinct entity, namely idiopathic hypereosinophilic syndrome (HES). A major challenge in reaching a definitive diagnosis was the unavailability of a biopsy-suitable lesion. The diagnostic algorithm is illustrated in Figs. 4 and 5 [6, 7].

Hypereosinophilic syndrome (HES) is a clinical condition characterized by excessive eosinophil production. The established diagnostic criteria include persistent eosinophilia (> 1500/μL) for at least 6 months, eosinophil-mediated target-organ damage, and the absence of other causes for hypereosinophilia. The clinical manifestations of HES vary depending on the extent of target-organ damage. Vasculitis is recognized as a distinct entity associated with HES, leading to the development of arterial and venous thrombosis. The occurrence of hypereosinophilic vasculitis stems from vascular damage resulting from either primary eosinophilic vasculitis or an underlying connective tissue disease. This condition predisposes patients to a prothrombotic state, increasing the risk of thrombosis formation in both veins and arteries [8]. However, it is worth noting that this condition has not yet been formally recognized as a separate entity in the International Chapel Hill Consensus Conference 2012 classification [9]. The peripheral symptoms, which were observed in this case, are also indicative of HES. Various forms of neuropathy have been documented, including multiple mononeuropathy, distal symmetrical motor neuropathy, and peripheral nervous system radiculopathy [10]. The precise mechanisms underlying eosinophil involvement in peripheral neuropathy remain unclear. It has been suggested that cationic protein, which was released from eosinophil cytotoxic granules, could play a partial role in thromboembolism, neurotoxicity, and major basic protein-mediated effects [10].

Hypereosinophilic vasculitis typically requires long-term treatment. Steroid therapy can lead to a rapid resolution of acute symptoms and maybe a life-saving intervention. The British Society of Rheumatology recommends the use of glucocorticoids in combination with intravenous pulse cyclophosphamide or rituximab to achieve disease remission in ANCA-associated vasculitis. Maintenance therapy is then recommended using azathioprine or methotrexate [11]. If patients are unable to tolerate or do not respond well to azathioprine or methotrexate, alternative options such as mycophenolate mofetil or leflunomide may be considered. Rituximab can also be utilized for maintenance treatment. The European League Against Rheumatism (EULAR), in collaboration with the European Renal Association and the European Vasculitis Society, suggests the consideration of plasma exchange for patients with rapidly progressive renal failure or pulmonary hemorrhage in newly diagnosed ANCA-associated vasculitis [12]. The presence of ANCA positivity can be identified in approximately 30–40% of individuals diagnosed with eosinophilic polyangiitis [13]. The presence or absence of ANCA can carry prognostic implications, with ANCA-negative individuals potentially experiencing poorer survival outcomes, likely due to a higher incidence of cardiac involvement [14].

However, ANCA-negative vasculitis is not specifically mentioned in these guidelines, indicating a lack of high-quality evidence, and most recommendations are based on expert opinions. Monitoring peripheral blood eosinophilia is a reasonable approach for monitoring these patients, although there is no clear correlation between eosinophil count and organ damage. Therefore, the patient’s response and symptoms remain the primary factors guiding decision-making until more specific and accurate markers of disease activity are introduced. In this case, the stable decrease of the absolute eosinophil could indicate a good response (Table 1).

## Conclusions

Eosinophilic vasculitis is an uncommon manifestation of hypereosinophilic syndromes and can be associated with thrombosis in both the arteries and veins. Our case study contributed information on this rare presentation and demonstrated the effectiveness of corticosteroid treatment.

## Limitation

The main limitation in this case is that the investigation of potential causes still remained incomplete due to the lack of skin biopsy confirmation. Besides, the genetic and Immunophenotyping tests to diagnose lymphocytic HES (L-HES) have not been done. This requires long-term follow-up for T-cell lymphoma.

## Data Availability

Not applicable.

## Abbreviations

ANCA: Antineutrophilic cytoplasmic antibody
CD40L: CD40 ligand
IL-4: Interleukin 4
FIP1L1/PDGFRA: Fip1-like 1-platelet-derived growth factor receptor alpha
HES: Hypereosinophilic syndrome
HIV: Human immunodeficiency virus
MRI: Magnetic resonance imaging
TAO: Thromboangiitis obliterans
TNF: Tumor necrosis factor
